# Metabolic Engineering Techniques to Increase the Productivity of Primary and Secondary Metabolites Within Filamentous Fungi

**DOI:** 10.3389/ffunb.2021.743070

**Published:** 2021-12-01

**Authors:** Koichi Tamano, Akira Yoshimi

**Affiliations:** ^1^Bioproduction Research Institute, National Institute of Advanced Industrial Science and Technology (AIST), Sapporo, Japan; ^2^Computational Bio Big-Data Open Innovation Laboratory (CBBD-OIL), National Institute of Advanced Industrial Science and Technology (AIST), Tokyo, Japan; ^3^Laboratory of Environmental Interface Technology of Filamentous Fungi, Graduate School of Agriculture, Kyoto University, Kyoto, Japan

**Keywords:** filamentous fungi, metabolite, metabolic engineering, gene knockout, gene overexpression

## Introduction

Filamentous fungi are multicellular eukaryotic microorganisms that primarily obtain energy *via* the degradation of macromolecules in their environment. The production of many of the hydrolytic enzymes required for feeding is prolific and the capacity has been exploited for the mass production of industrially relevant enzymes such as amylase, protease, and lipase. Fungi can also produce large quantities of chemicals. In 1917, *Aspergillus niger* was found to have the capacity to produce citrate, a primary metabolite, that is now ubiquitously used as an acidulent (Cairns et al., 2018). In 1928, *Penicillium rubens* was found to produce penicillin, a secondary metabolite exhibiting antibacterial activity (Browne et al., 2013). In the intervening decades, filamentous fungi have been intensively researched to identify new chemicals, of which many are widely used as pharmaceuticals (e.g., cyclosporin and lovastatin) (Boruta, 2018), pigments (e.g., Monascus pigment) (Liu et al., 2018), platform chemicals (e.g., itaconic acid) (Huang et al., 2021), and cosmetics (e.g., kojic acid) (Terabayashi et al., 2010).

Industry has used a variety of microbial hosts including bacteria (e.g., *Escherichia coli, Streptomyces* sp., *Corynebacterium* sp.), yeasts (e.g., *Saccharomyces cerevisiae, Pichia pastoris, Yarrowia lipolytica*), and filamentous fungi to produce valuable metabolites. Among them, filamentous fungi have considerable merits that are ability to degrade polymers, availability in solid-state cultures, and suitability to produce key primary and secondary metabolites (Abe et al., 2006; Machida et al., 2008).

In recent years, next-generation DNA sequencing (NGS) technology has been widely used for genome and transcriptomic analysis (Goodwin et al., 2016). Noteworthy, NGS devices enabled high-throughput sequencing at a much lower cost as compared to conventional Sanger sequencing (Reuter et al., 2015). The impact of post-sequencing analytical tools to precisely annotate genes cannot be understated (Gene Ontology Consortium, 2021). For example, industrial strains of filamentous fungi other than type strains have been subjected to genome sequencing by NGS and the specific genes involved in the biosynthesis of many target metabolites can now be predicted in industrial strains. Thus, technology based on genomic information has in fact become applicable to industrial strains. Furthermore, enhancing industrial productivities of target metabolites by metabolic engineering can be attained.

However, there are still several limitations to industrial utilization. Based on author's experimental experience of filamentous fungi *Aspergillus* sp., for example, it is difficult to obtain high-density liquid cultures because of pellet formation (Miyazawa et al., 2016). Additionally, laborious experiments are required for their genetic manipulation compared to other host microorganisms. For several strains, single spore isolation is necessary for mutant construction due to their multinuclear sporulation characteristics (Maruyama et al., 2002). High-throughput screening is also difficult owing to large colony formation. The time taken to construct mutants *via* recombination is longer because several days are required for spore formation using agar culture. Therefore, research aiming to overcome these drawbacks has been performed for many years. Several progresses have been reported and are briefly introduced at the next two sections.

## Recombination Technologies

In filamentous fungi, the integration of transforming DNA by the non-homologous end joining (NHEJ) pathway occur at a much higher frequency than by the homologous recombination (HR) pathway (Takahashi et al., 2004). Thus, DNA fragments taken up by nuclei end up being integrated at random locations in the genome rather than at specific, targeted locations. Since most strategies for strain improvement require targeted integration of DNA, NHEJ is a hindrance. However, in 2004 and 2006, genetic factors involved in the NHEJ pathway were identified in *Neurospora crassa* (Ninomiya et al., 2004; Ishibashi et al., 2006). Following this identification, mutation of NHEJ-involved genes such as *ku70, ku80*, and *ligD* that were conserved among many fungal species created strains whereby subsequent DNA integrations occurred mostly by HR rather than NHEJ. This increased efficiency of HR (exceeding 90% in some filamentous fungi) vastly improved strain breeding pipelines (Takahashi et al., 2006; Mizutani et al., 2008).

The constructed NHEJ-knockout strain can be utilized for metabolic engineering. For metabolic engineering, the pipeline for strain breeding generally includes deletion of a single or multiple genes and gene overexpression experiments. To remove unnecessary metabolic pathways to allow target metabolite overproduction, it is fundamental to inactivate the first reaction of unnecessary pathways by deleting a key enzyme-coding gene (Tamano, 2014). Target metabolite biosynthesis may also be facilitated by overexpression of a bottleneck enzyme-coding gene (Wong et al., 2021). In this case, the original promoter is changed to a promoter from a constitutively highly expressed gene. For gene deletion, target gene is replaced with a selectable marker by two HR events between chromosome and introduced DNA (e.g., double HR) (Tamano et al., 2015; Wendt et al., 2016); for gene overexpression, target gene promoter is replaced with a set of selectable marker and constitutive high-expression promoter by double HR (Tamano et al., 2013). Gene overexpression can alternatively be attained by transforming with a plasmid harboring the overexpression cassette (Kanamasa et al., 2001). In this case, a single HR event is sufficient for the cassette to be inserted into the chromosome.

Recently, the newly discovered editing technology, CRISPR/Cas9, has been applied to modify fungal genomes (reviewed in Jiang et al., 2021). In this case, gene disruption and overexpression occur *via* a targeted double strand DNA break followed by genome editing. For gene disruption, a selectable marker is introduced into the target gene; for gene overexpression, a DNA cassette composed of a selectable marker and constitutive high-expression promoter is introduced to a locus just upstream of the start codon of the gene.

## Selectable Markers and Promoters

Selectable marker genes must be available for mutant construction for metabolic engineering. They are categorized into three groups: [1] antifungal resistance, [2] auxotrophy restoration, and [3] nutrient-catabolizing. Five genes commonly used to confer resistance to different drugs (pyrithiamine, hygromycin, bleomycin, aureobasidin, and oligomycin) are *ptrA, hph, ble, aur*, and *oliC31* (Gritz and Davies, 1983; Ward et al., 1986; Glumoff et al., 1989; Heidler and Radding, 1995; Kubodera et al., 2000). Three genes commonly used to restore auxotrophy are *pyrG, niaD*, and *sC* (van Hartingsveldt et al., 1987; Unkles et al., 1989; Yamada et al., 1997). *amdS* is the lone nutrient-catabolizing enzyme gene commonly used (Gomi et al., 1991). Some selectable markers can be “reused” to allow sequential engineering events through a process called counterselection. For example, the portion of a DNA cassette, *pyrG*, conferring pyrimidine prototrophy integrated into a chromosome by HR can be removed *via* DNA loop-out or by Cre recombinase coupled with *loxP* recognition sites under existence of 5-fluoroorotic acid (Zhang et al., 2017). Thus, using this marker-recycling system, multiple mutation events are possible in a single genome with essentially no limitation. By combining the use of these selectable markers, strains genetically engineered at multiple genomic locations can be constructed.

Additionally, high-expression promoters have to be utilized for gene overexpression. The translation elongation factor 1 (*tef1*)-encoding gene is constitutively expressed at high levels in *Aspergillus oryzae* (Kitamoto et al., 1998). Because this gene and promoter are highly conserved in distantly related fungi, the level of gene expression in heterologous hosts should be similar. Moreover, genes encoding glycolytic enzymes such as enolase are constitutively expressed at high levels. Promoters of these genes are usually utilized for gene overexpression. Recently, Jin et al. (2021) provided a comprehensive review of selectable markers and promoters commonly used to genetically engineer *Aspergillus oryzae*.

## Outline of Industrial Strain Breeding of Filamentous Fungi: From Identification to Metabolic Engineering

**Figure 1:**
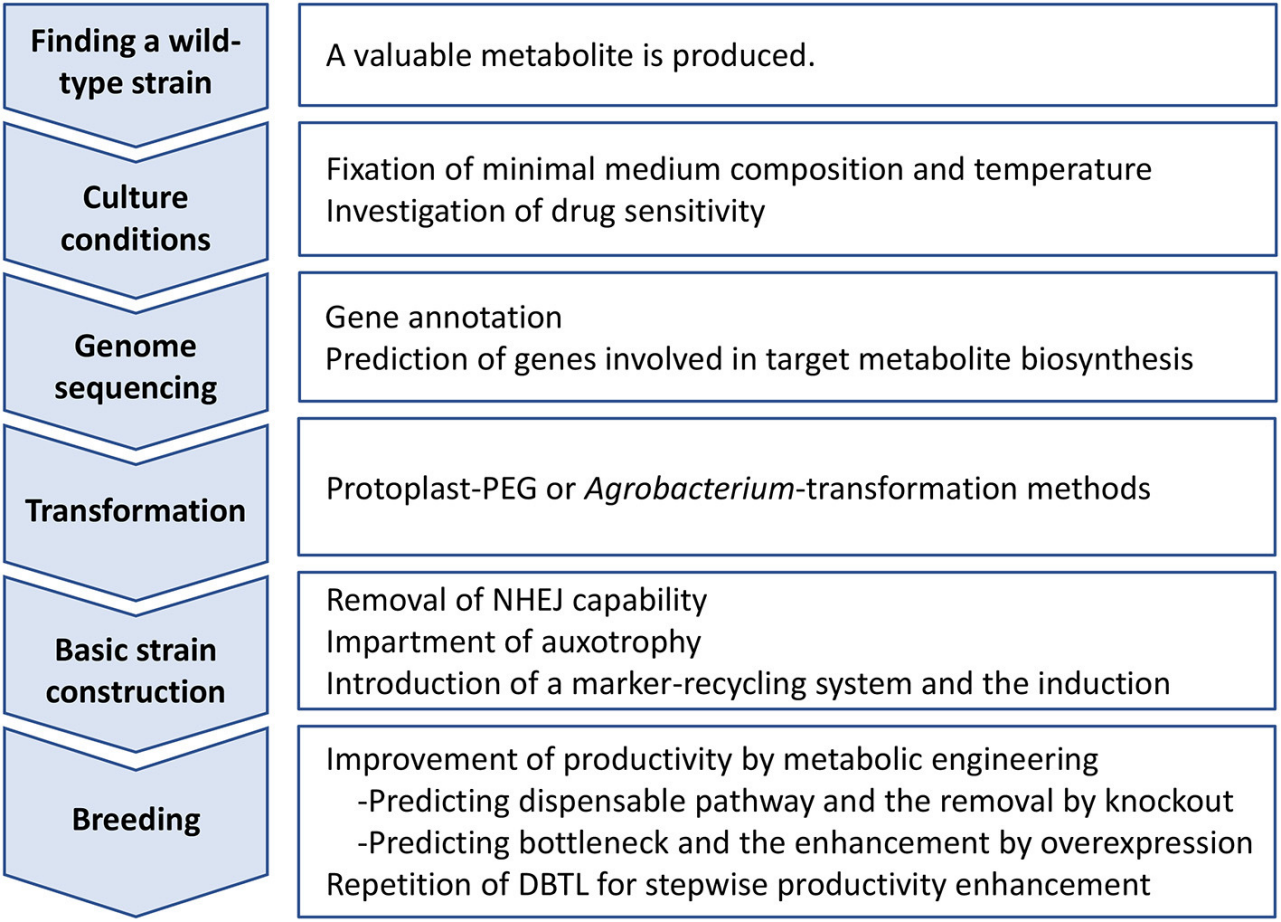
Outline of industrial strain improvement of filamentous fungi for enhancing productivity of a target metabolite. DBTL, Design-Build-Test-Learn cycle; NHEJ, non-homologous end joining; PEG, polyethylene glycol.

Process of improving industrial strains by metabolic engineering, as an opinion of authors, is outlined in Figure 1. The first step of the process is to identify a wild-type strain that produces a valuable metabolite. Large-scale screening of filamentous fungal strains is necessary for the identification. However, it does not promise successful identification of such strains. Therefore, this step is most challenging. Next, as the second step, establishing adequate culture conditions is critical, especially defining the minimal medium composition because auxotrophic selectable markers may be used in subsequent strain improvement efforts. Similarly, as drug-resistant selectable markers may be considered for strain improvement, it is important to investigate drug sensitivity. The third step is NGS-based genome sequencing, gene annotation, and whole-genome transcriptional analysis. The latter transcriptional analysis can be particularly helpful if culture conditions conducive to product formation are established. The comparison of gene transcription after growth on metabolite production media vs. non-production media can contribute significantly to the accuracy of predicting genes involved in the biosynthesis of the target metabolite. The step requires NGS sequencing, and thus seems most expensive. The fourth step is to construct a genetic transformation system of the wild-type strain identified. Polyethylene glycol-mediated transformation protocol using protoplasts is very common and has been developed for a wide range of filamentous fungi (Tamano et al., 2007). This step requires trial and error to find an optimal condition of protoplast formation. Cell wall-lysing enzymes and the mixed composition must be optimized. Hence, it is labor-intensive. If protoplasts cannot be prepared in sufficient amounts to be used for transformation, a number of *Agrobacterium*-mediated transformation protocols have been developed (Takahashi et al., 2011). The fifth step is the construction of a basic strain for metabolic engineering. Briefly, genes *ku70, ku80*, and *ligD* responsible for NHEJ are extracted by homology search and then one of them is mutated by low-efficiency double HR with use of a drug-resistant selectable marker. The loss of NHEJ will allow acquisition of subsequent HR-mediated mutant strains at high-efficiency. After removing NHEJ capacity, a gene involved in essential metabolism is knocked out by double HR with another drug-resistant selectable marker. For example, *pyrG* gene, which partakes in the pyrimidine biosynthesis, is comparatively short and completely lethal when knocked out; therefore, it is a good candidate of knockout as well as an auxotrophic selectable marker (van Hartingsveldt et al., 1987). A marker-recycling system of Cre/*loxP* is then introduced to an appropriate locus on the chromosome with use of auxotrophic selectable marker enabled just before. At this point, the DNA fragment is designed as the Cre recombinase gene expression can be induced specifically under certain culture conditions. For instance, a xylanase gene promoter is placed ahead of the Cre-coding sequence; thus, Cre is only expressed when xylose is used as a carbon source in the culture (Zhang et al., 2017). After induction of the Cre/*loxP* system, an auxotrophic strain in which the NHEJ pathway is defective and the marker-recycling system reside is consequently constructed *via* counter-selection using 5-fluoroorotic acid. It is the basic strain for breeding. Lastly, as the sixth step, the metabolic engineers can now work directly to enhance target metabolite synthesis. A first common target is to identify and disable dispensable metabolic pathways that would consume primary feedstocks essential for target metabolite synthesis. The first reaction of a dispensable metabolic pathway competing with target metabolite biosynthesis is knocked out by deletion of the associated metabolic enzyme gene. Next, engineers focus on identifying “bottleneck” enzyme reactions that limit target metabolite biosynthesis predicted by analyzing transcriptomic and metabolomic data. One common solution to overcome a bottleneck is to overexpress the rate limiting enzyme-coding gene (Tamano, 2014). The relief of one bottleneck often uncovers secondary bottlenecks, which requires the process to be repeated. That is, repetition of workflow composed of prediction of bottleneck reaction *via* analysis of transcriptomic and metabolomic data, and subsequent cancellation of the bottleneck by metabolic engineering. This manner of productivity enhancement involving repeated workflow is referred to as the Design-Build-Test-Learn (DBTL) cycle (Vavricka et al., 2020).

## Future Prospects

Genomic information coupled with new techniques to genetically engineer filamentous fungi has led to tremendous progress in strain improvement in recent years. Hereafter, the development of new techniques focused on increasing efficiency and moving to high throughput mutant construction is required. Moreover, the application of flux balance analysis based on metabolism modeling and other multi-omics analysis will allow engineers to more accurately identify bottleneck genes. Continued progress in development of new experimental microbiology and molecular biology techniques as well as bioinformatic tools related to metabolic engineering, is required to attain the efficient productivity improvements needed by society up until now and from now on.

## Author Contributions

KT drafted the opinion text. AY checked the draft. All authors contributed to the article and approved the submitted version.

## Conflict of Interest

The authors declare that the research was conducted in the absence of any commercial or financial relationships that could be construed as a potential conflict of interest.

## Publisher's Note

All claims expressed in this article are solely those of the authors and do not necessarily represent those of their affiliated organizations, or those of the publisher, the editors and the reviewers. Any product that may be evaluated in this article, or claim that may be made by its manufacturer, is not guaranteed or endorsed by the publisher.
